# The prevalence of obesity and hypertension in paediatric cardiology patients before and after COVID-19 lockdown measures

**DOI:** 10.1007/s00431-026-06816-7

**Published:** 2026-02-25

**Authors:** Jemima Ball, Jasmine Hulme Kenny, Charalampos Kotidis

**Affiliations:** East Midlands Congenital Heart Centre, University Hospitals of Leicester, Leicester, UK

**Keywords:** Paediatric cardiology, COVID-19, Obesity, Hypertension, Prevention

## Abstract

This study is a retrospective clinical audit to evaluate the impact of COVID-19 lockdown measures on the prevalence of obesity and hypertension in paediatric cardiology patients and determine the extent to which these comorbidities were recognised and managed at the East Midlands Congenital Heart Centre (EMCHC), UK. Height, weight and blood pressure (BP) values were extracted from clinic letters before and after COVID-19 lockdown at the EMCHC. BMI and BP percentiles were calculated to categorise BMI and BP stage. Analysis compared pre- and post-lockdown outpatient clinic data of paediatric cardiology patients. 800 patients were included. Mean BMI increased from 17 to 20 kg/m^2^, with a 3% rise in obesity prevalence. South Asian children were the only ethnic group to show a significant post-lockdown increase in BMI percentile. Patients with severe congenital heart disease (CHD) had lower BMI than those with mild or repaired lesions. Although systolic and diastolic BP percentiles declined significantly post-lockdown (*P* < 0.001), 29% of patients met thresholds for stage 1 or stage 2 hypertension, likely an overestimate due to single automated readings and white-coat effects. BMI correlated positively with systolic BP in both periods (pre-lockdown *r* = 0.164; post-lockdown *r* = 0.297). Only 2% of hypertensive patients and 2% of patients with obesity were appropriately referred for further management. *Conclusion*: Obesity and hypertension remain under-recognised and undertreated in paediatric cardiology patients. Strong BMI–BP associations underscore the need for repeated manual BP readings, ambulatory monitoring and routine use of centile-based assessment to optimise long-term cardiovascular outcomes.

**Table:** 

**What is Known:** • *Childhood obesity and hypertension have increased globally, worsened by COVID-19 lockdowns.* • *Paediatric cardiac patients are especially vulnerable due to limited cardiac reserves and accelerated cardiovascular ageing.* • *These risks are often under-recognised in clinics.*	
**What is New:** • *Post-lockdown BMI and obesity rose particularly in South Asian children.* • *Hypertension was common yet seldom documented or acted upon.* • *Strong BMI–systolic BP correlations, disease-severity differences and missed referrals emphasise the need for percentile use, repeat BP checks and better integrated cardiometabolic pathways in paediatric cardiology.*

**What is Known:**

• *Childhood obesity and hypertension have increased globally, worsened by COVID-19 lockdowns.*

• *Paediatric cardiac patients are especially vulnerable due to limited cardiac reserves and accelerated cardiovascular ageing.*

• *These risks are often under-recognised in clinics.*

**What is New:**

• *Post-lockdown BMI and obesity rose particularly in South Asian children.*

• *Hypertension was common yet seldom documented or acted upon.*

• *Strong BMI–systolic BP correlations, disease-severity differences and missed referrals emphasise the need for percentile use, repeat BP checks and better integrated cardiometabolic pathways in paediatric cardiology.*

## Introduction

COVID−19 lockdown measures resulted in profound lifestyle disruption for children and adolescents. Reduced opportunities for physical activity, cancellation of organised sports, prolonged indoor confinement and significant increases in sedentary behaviour led to rapid rises in childhood obesity across international populations [1–5]. These trends are concerning because childhood obesity and hypertension are strong predictors of premature cardiovascular disease, with long-term cohort studies demonstrating clear associations with myocardial infarction, stroke and early mortality [6–8].

Children with congenital heart disease (CHD) are particularly vulnerable to these risks. Many exhibit features of accelerated cardiovascular ageing, including increased arterial stiffness, endothelial dysfunction and limited physiological reserve, even in childhood [9]. For such children, increases in weight or blood pressure may exert disproportionately harmful effects on ventricular workload, haemodynamics and long-term cardiovascular resilience.

Despite these concerns, little is known about obesity and hypertension trends in paediatric cardiology populations, particularly in relation to COVID-19 lockdowns. Most existing literature examines children without CHD, often focusing on populations with obesity, hypertension, dyslipidaemia or other systemic conditions, leaving a significant gap regarding cardiometabolic risk in paediatric cardiology populations, who may be particularly vulnerable to lifestyle disruption.

This audit aimed to describe the prevalence of obesity and hypertension before and after COVID-19 lockdowns in paediatric cardiology patients at East Midlands Congenital Heart Centre (EMCHC), UK. It further sought to assess demographic and clinical associations and to evaluate whether abnormal findings were adequately recognised and managed in practice.

## Methods

### Study design and population

A retrospective clinical audit was conducted among paediatric cardiology patients at the East Midlands Congenital Heart Centre (EMCHC), University Hospitals of Leicester (UHL), UK. Data were collected for the same cohort of patients who attended outpatient clinics before and after the COVID-19 lockdown period. Eligible participants were aged 2–18 years, were at least 2 years old during their pre-lockdown visit (before March 2020) and had not reached 18 years by March 2025. Pre-lockdown data were collected between March 2017 and March 2020 and post-lockdown data between January 2022 and March 2025.

#### Data collection

At each outpatient visit, weight, height and BP were measured by trained healthcare assistants before the medical review and documented in the clinic letter. Patients were identified via the hospital’s electronic cardiology database.

BMI and BP percentiles and Z-scores were calculated using Body Composition Laboratory calculators [10, 11]. Body mass index (BMI) categories were defined using age- and sex-specific BMI-for-age percentiles in accordance with established paediatric growth standards [12]. Blood pressure (BP) categories were defined using percentile-based thresholds adjusted for age, sex and height, in line with international paediatric hypertension guidelines [13]. Full definitions and thresholds are provided in Appendix 1.

#### Cardiac disease classification

CHD severity was classified using clinically recognised categories aligned with American Heart Association complexity definitions:

- No established cardiac phenotype: patients under ongoing paediatric cardiology surveillance due to recognised cardiac risk or symptoms, including family history of inherited cardiac conditions, prior exposure to cardiotoxic chemotherapy, connective tissue disorders or recurrent palpitations, chest pain and/or syncope, in whom a definitive structural or arrhythmic diagnosis has not yet been established.
- Arrhythmia: Primary rhythm abnormalities or inherited arrhythmic syndromes without structural congenital heart disease, including atrioventricular block, channelopathies and other conduction or rhythm disorders requiring ongoing cardiology follow-up.
- Mild CHD: Structural cardiac abnormalities that are asymptomatic or minimally symptomatic, including small shunt lesions and mild valve stenosis or regurgitation managed with clinical surveillance alone.
- Moderate CHD: Haemodynamically significant structural abnormalities, including valve or shunt lesions, requiring ongoing medical therapy and/or catheter-based or surgical intervention.
- Severe CHD: Complex congenital cardiac lesions requiring early or repeated surgical intervention, including single-ventricle physiology (e.g. Fontan circulation), cyanotic congenital heart disease (e.g. transposition of the great arteries, tetralogy of Fallot), major outflow tract reconstructions or other high-complexity abnormalities.

Socioeconomic status was determined using postcode-derived index of multiple deprivation (IMD) deciles [14]. IMD ranks residential areas in the UK based on seven domains: income, employment, education, health and disability, crime, barriers to housing and services and living environment. Decile 1 represents the most deprived areas, and decile 10 is the least deprived areas. Lower IMD deciles therefore reflect greater socioeconomic vulnerability.

#### Statistical analysis

Analyses were performed using SPSS (version 29). Continuous variables were summarised as mean (SD) and compared using Student’s *t*-tests. Associations were assessed with Spearman’s correlation and categorical variables with chi-square tests. Statistical significance was defined as *P* < 0.05.

## Audit standards

Local University Hospitals of Leicester (UHL) guidelines were used to assess whether the management of obesity and hypertension, including referrals to the Complications of Excess Weight (CEW) clinic and paediatric hypertension specialists, was appropriate (Appendix 2).

## Results

A total of 800 paediatric cardiology patients were included. The mean (SD) age in the pre-lockdown period was 6.9 (3.6) years; 561 (70%) were White British and 461 (58%) were male. Further demographic and clinical characteristics are summarised in Table 1.

**Table 1:** Demographics and clinical outcomes of paediatric cardiology patients before and after lockdown measures. Data are presented as mean, standard deviation and percentage as appropriate

Patients (*N*)	Pre-COVID	Post-COVID	*P* value
	800		
White British South Asian Black Mixed Other	561 (70%) 127 (16%) 11 (1.5%) 27 (3.5%) 74 (9%)		
Male	461 (58%)		
IMD rank	16,201 (9754)		
IMD decile	5 (3)		
No cardiac phenotype Arrhythmias Mild CHD Moderate CHD Severe CHD	69 (8.5%) 64 (8%) 299 (37.5%) 119 (15%) 249 (31%)		
Age (years)	6.9 (3.6)	12 (3.8)	< 0.001
Weight (kg)	25 (13)	44 (20)	< 0.001
Height (m)	1.17 (0.24)	1.44 (0.21)	< 0.001
BMI (kg/m^2^)	17 (3.1)	20 (5.4)	< 0.001
BMI Z score	0.04 (1.32)	0.25 (1.34)	< 0.001
BMI percentile	52 (33)	57 (32)	< 0.001
Weight profile Underweight Healthy weight Overweight Obese Severely obese	65/676 (9%) 451/676 (67%) 89/676 (13%) 67/676 (10%) 4/676 (1%)	60/800 (8%) 518/800 (65%) 107/800 (13%) 104/800 (13%) 11/800 (1%)	0.267
BP staging Normal Elevated Stage 1 HTN Stage 2 HTN	352/688 (51%) 103/688 (15%) 173/688 (25%) 60/688 (9%)	431/791 (54%) 135/791 (17%) 180/791 (23%) 45/791 (6%)	0.004
SBP percentile	78 (23)	71 (26)	< 0.001
DBP percentile	68 (24)	65 (26)	< 0.001

*BMI* body mass index, *BP* blood pressure, *HTN* hypertension, *IMD* index of multiple deprivation, *SBP* systolic blood pressure, *DBP* diastolic blood pressure

As expected in a longitudinal cohort, mean weight and height increased between pre- and post-lockdown assessments. Correspondingly, mean BMI was significantly increased from 17 to 20 kg/m^2^ over the study period (*P* < 0.001). BMI percentile significantly increased from 52 to 57 (*P* < 0.001). Analysis of BMI categories showed a small reduction in the proportion of underweight (− 1%) and healthy-weight (− 2%) patients, while the proportions in the overweight and severely obese categories remained stable. By contrast, the proportion of patients classified as having obesity increased by 3%. This shift in weight profiles did not reach statistical significance when analysed across all BMI categories (*P* = 0.267). Among the 115 patients classified as having obesity or severe obesity in the post-lockdown period, obesity was documented as a comorbidity in clinic correspondence for only six patients, highlighting substantial under-recognition.

In contrast to BMI trends, blood pressure staging differed significantly between pre- and post-lockdown assessments when analysed across all four categories (normal, elevated blood pressure, stage 1 and stage 2 hypertension; *P* = 0.004; Table 1). This reflected a redistribution within categories, characterised by a reduction in stage 2 hypertension and a corresponding increase in normal and elevated blood pressure classifications. Consistent with this, both systolic and diastolic blood pressure percentiles declined significantly after lockdown (*P* < 0.001 for both). However, when hypertension was analysed as a binary outcome, the proportion of patients meeting criteria for stage 1 or stage 2 hypertension remained similar between periods (233 pre-lockdown vs 225 post-lockdown). Despite this, hypertension was documented as a comorbidity in clinic letters for only nine patients (4%) in the post-lockdown period.

**Fig. 1:**
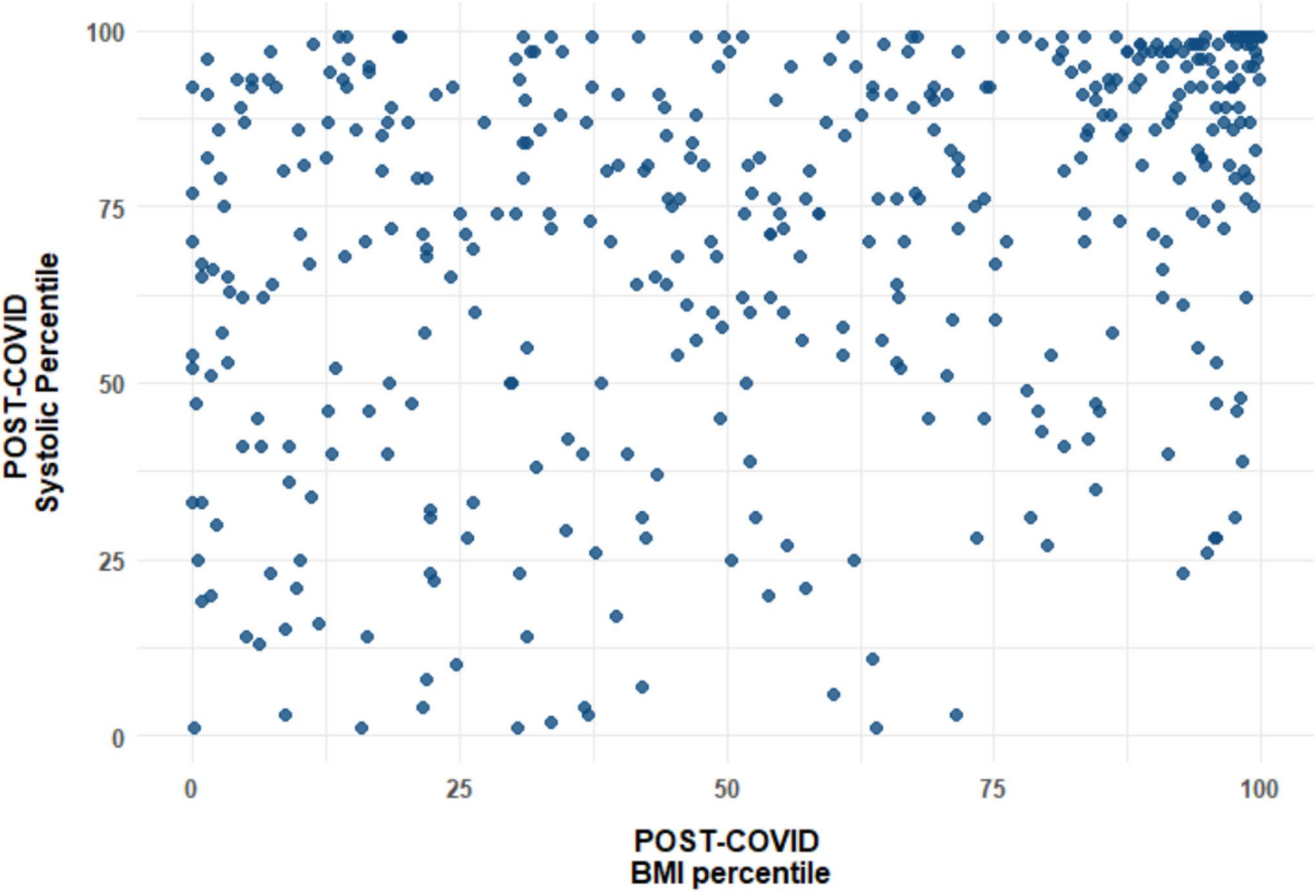
Scatter plot depicting the relationship between post-COVID systolic blood pressure and post-COVID BMI percentiles. While BMI percentiles are widely distributed across the sample, systolic blood pressure percentiles show clustering at higher values with higher BMI percentiles. Graphics drawn using SPSS (version 29)

Spearman’s correlation analysis demonstrated a significant positive association between BMI and systolic BP percentiles in both time periods (pre-lockdown *r* = 0.164, *P* < 0.001; post-lockdown *r* = 0.297, *P* < 0.001 (Fig. 1). This association was stronger post-lockdown, suggesting an increasing contribution of excess weight to systolic BP over time. No significant correlation was found between BMI and diastolic BP percentiles in either period.

**Fig. 2:**
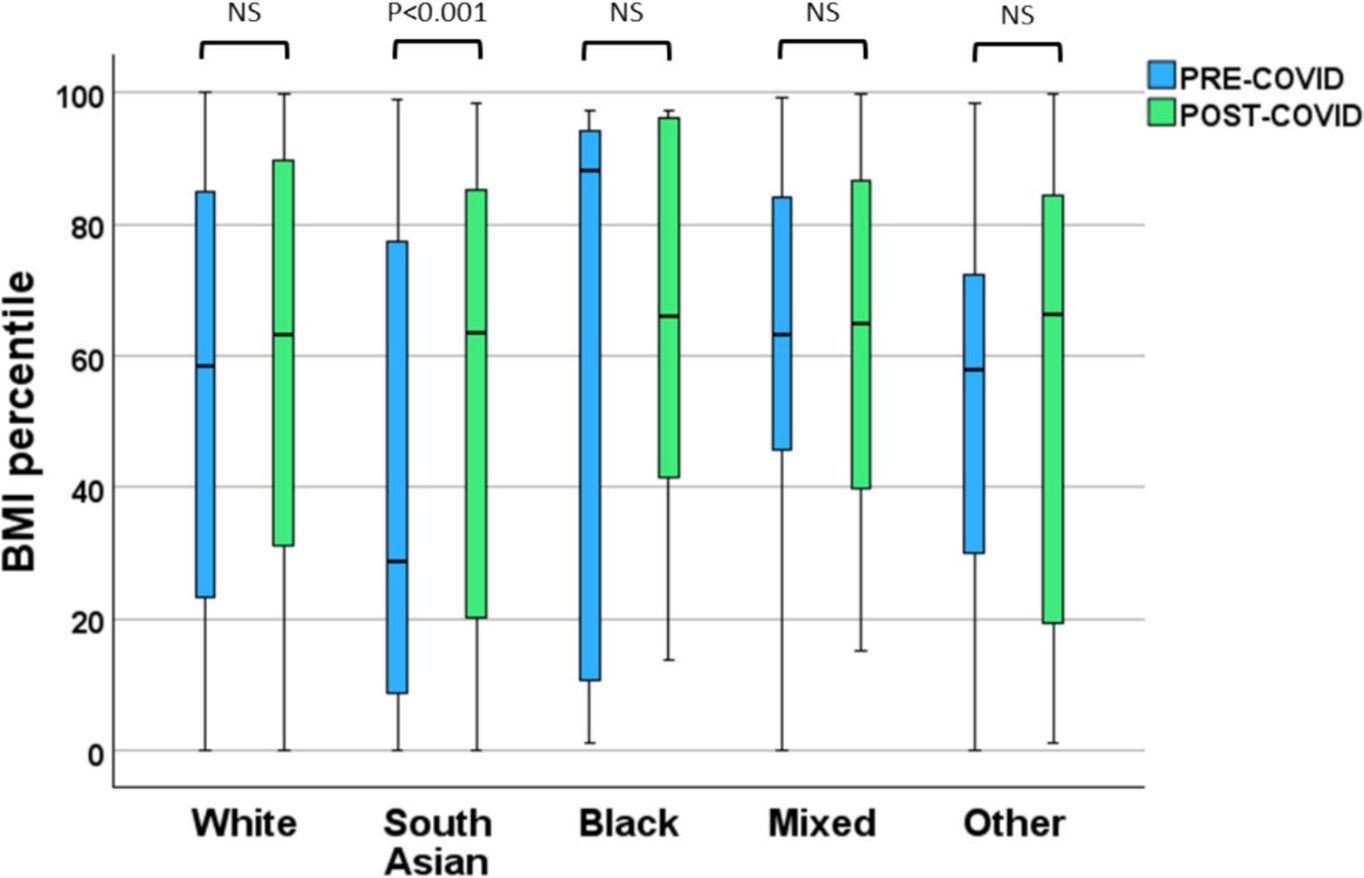
Comparison of BMI percentiles before (blue) and after (green) the COVID-19 pandemic across different ethnic groups. Significant differences between pre- and post-COVID groups were observed only in the South Asian cohort (*P* < 0.001), while no significant differences (NS) were found in White, Black, mixed or other groups. Graphics drawn using SPSS (version 29)

**Fig. 3:**
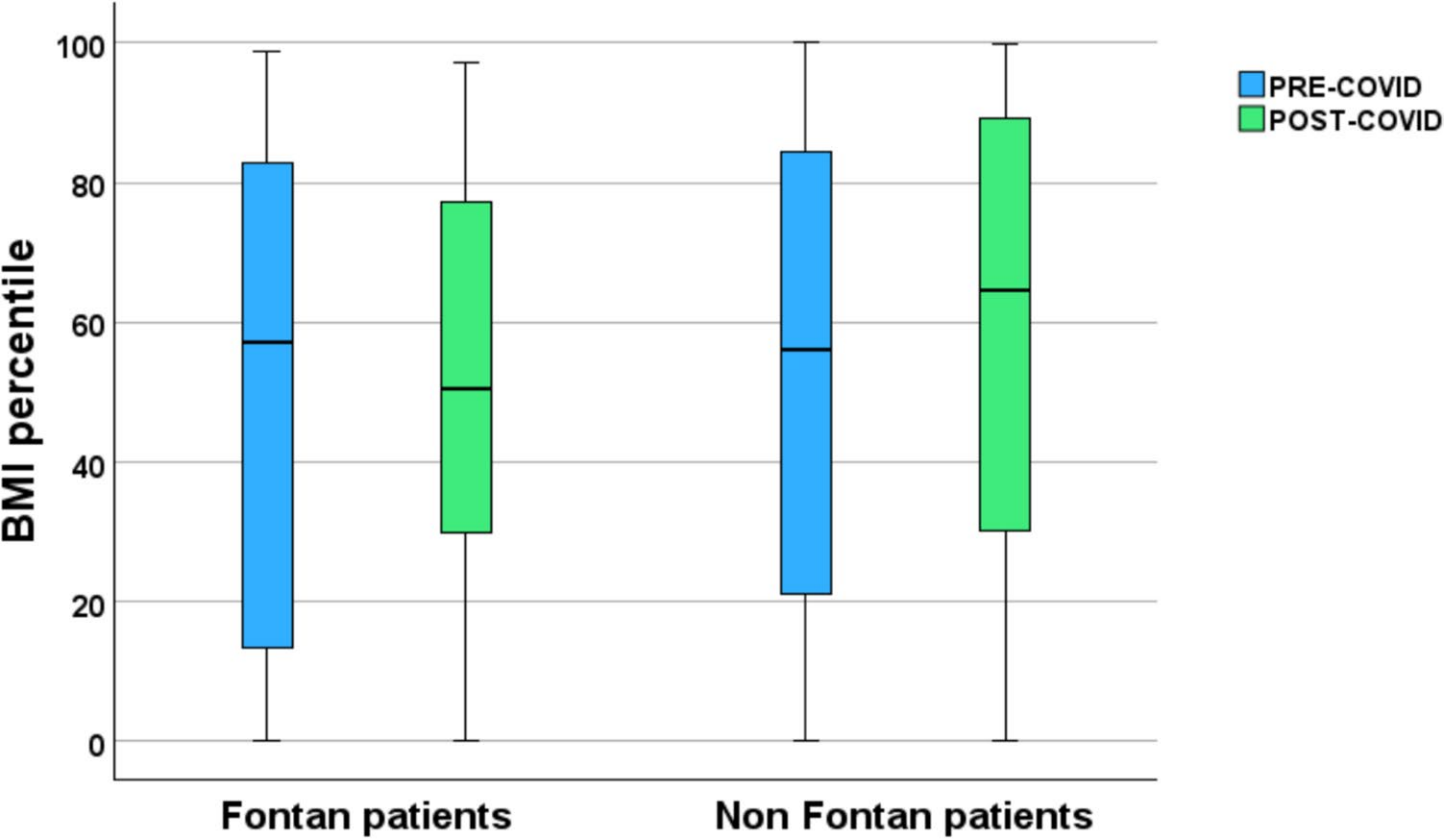
Comparison of BMI percentiles in Fontan and non-Fontan patients before (blue) and after (green) the COVID-19 lockdown periods. Graphics drawn using SPSS (version 29)

There was no significant association between change in BMI percentile and age, sex or IMD decile. When stratified by ethnicity, only patients of South Asian background showed a statistically significant increase in BMI percentile following lockdown measures (Fig. 2). Subgroup analysis of patients with Fontan circulation (*n* = 44) revealed a small, non-significant increase in BMI percentile following lockdown (median [IQR] 50 [34] vs 52 [30]). By comparison, non-Fontan patients exhibited a significant increase in BMI percentile over the same period (52 [32] vs 58 [32] kg/m^2^; *P* < 0.001) (Fig. 3).

**Fig. 4:**
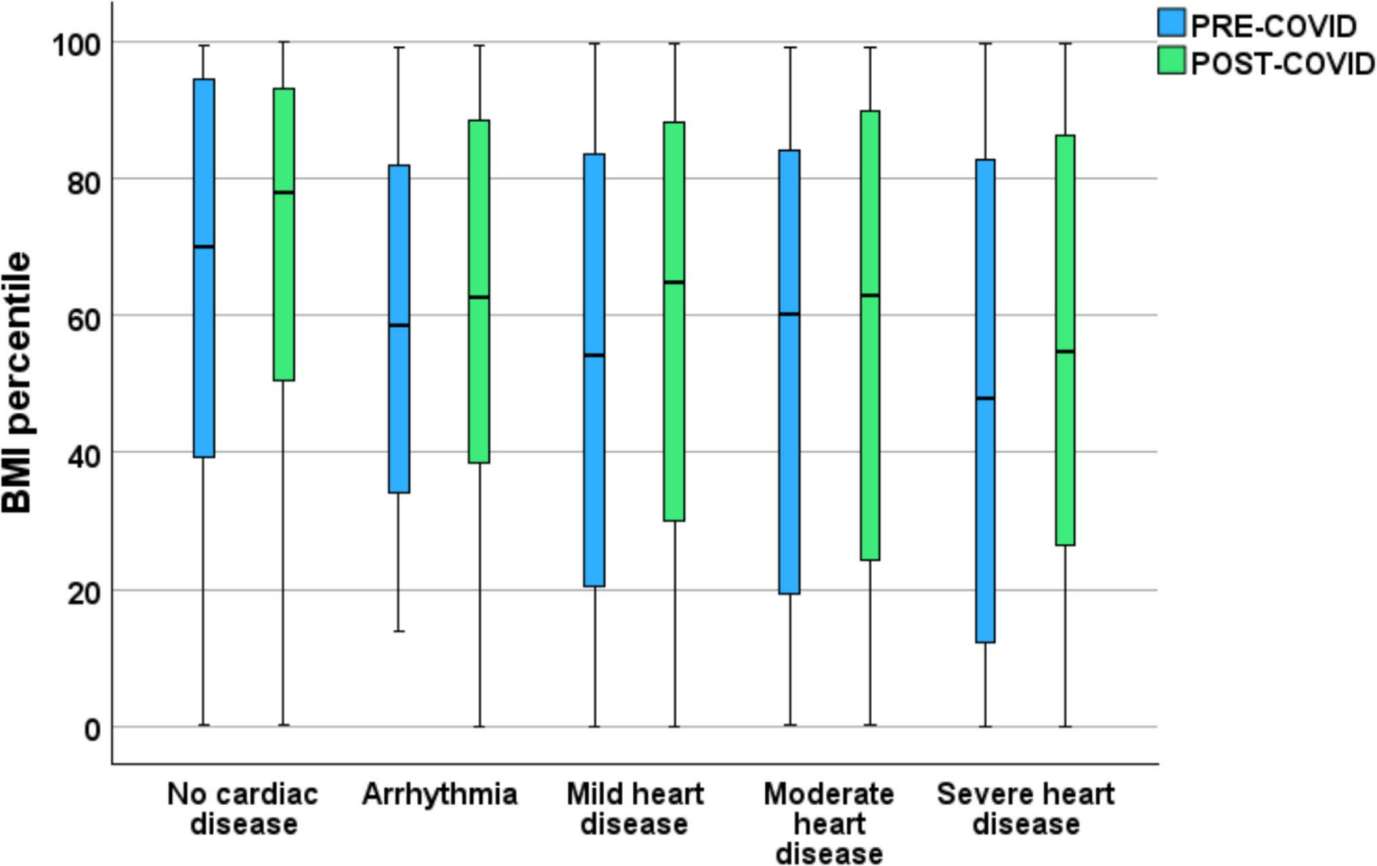
Comparison of BMI percentiles before (blue) and after (green) the COVID-19 pandemic among patients based on disease severity. Across groups, median BMI percentiles tended to be lower with increasing severity of cardiac disease. Graphics drawn using SPSS (version 29)

When patients were grouped by cardiac disease severity, a progressive decrease in BMI percentile was observed with increasing severity in both pre- and post-lockdown data. Those with severe CHD had the lowest median BMI percentile (Fig. 4).

## Management and referral patterns

Applying UHL guidelines identified seven patients eligible for CEW referral; only two were previously referred. Extrapolating to annual EMCHC activity suggests an additional 39 patients per year would qualify if CHD were recognised as a referral criterion.

Of 225 hypertensive patients, only 5 (2.2%) received specialist management. Extrapolated, approximately 1733 annual EMCHC patients would meet hypertension thresholds. Guideline-recommended follow-up with ambulatory blood pressure monitoring (ABPM) occurred infrequently, even among high-risk CHD groups (patients with previous coarctation and/or arch hypoplasia).

## Discussion

### Summary of findings

This audit shows that obesity and high BP readings are common yet under-recognised and undertreated in paediatric cardiology patients. BMI percentiles increased significantly after lockdown measures, with a clinically relevant 3% rise in obesity, and this increase was particularly pronounced among South Asian children. Stage 1 and 2 hypertension was recorded in approximately one-third of patients across the study period, but very few received appropriate investigation or specialist follow-up. Importantly, these abnormalities were rarely documented as active problems in clinic letters, highlighting missed opportunities for intervention. To our knowledge, this is the first UK study to examine obesity and hypertension in paediatric cardiology patients before and after COVID-19 lockdown measures, and it provides novel data on ethnic disparities and the interaction between CHD severity, BMI and BP.

### Interpretation of obesity trends

Although the increase in obesity prevalence did not reach statistical significance when analysed across multiple BMI categories, the observed BMI percentile increase is clinically important in a group already at elevated cardiovascular risk. Patients with CHD are more vulnerable to the adverse haemodynamic and metabolic effects of excess weight than healthy peers, and early life obesity and hypertension are known to predict premature cardiovascular events and mortality in adulthood [6–8, 15]. Even modest BMI increases in childhood can translate into a higher lifetime burden of CVD, particularly when superimposed on structural heart disease.

Ethnic differences in BMI trajectories were observed following lockdown. Although Black children had the highest absolute BMI percentiles both before and after the pandemic, only South Asian children demonstrated a statistically significant increase in BMI percentile over time. This finding is consistent with UK-based evidence showing heightened cardiometabolic vulnerability among South Asian populations and disproportionate impacts of COVID-19 in these communities [16, 17]. Potential contributing factors include higher baseline risk of insulin resistance, socioeconomic disadvantage, environmental stressors and a greater likelihood of living in multi-generational households, which may have prompted more cautious adherence to lockdown measures and reduced physical activity in children. The apparent reduction in BMI percentile among Black children should be interpreted cautiously, as this subgroup was small and therefore underpowered to support reliable inference regarding temporal trends.

The WHO Asia–Pacific classification recognises that South Asian adults have lower BMI thresholds for obesity and cardiometabolic risk compared with White European populations [18]. Although paediatric BMI percentiles are used across ethnicities, an upward shift in BMI during adolescence in South Asian children may therefore represent a particularly concerning trajectory for future cardiac and metabolic complications. These findings underscore the need for tailored prevention and intervention strategies that account for ethnic background, cultural context and social determinants of health.

Emerging longitudinal evidence further supports the interpretation that BMI percentile increases in our cohort represent meaningful adiposity gain rather than simple ageing. A large, multicentre prospective study by Bichteler et al. demonstrated that children who followed “high-rising” or persistently elevated BMI percentile trajectories from early childhood to adolescence subsequently had significantly higher adolescent BMI, greater waist circumference and higher rates of elevated or pre-hypertensive blood pressure at age 15 compared with peers with stable trajectories [13]. Importantly, these adverse cardiometabolic profiles were driven by trajectory patterns, not chronological age alone, indicating that increases in BMI percentile, especially during sensitive developmental periods, are prognostically significant. When considered alongside our findings, this suggests that the 3% rise in obesity and the upward BMI percentile shift post-lockdown likely reflect true worsening of weight status rather than the expected effects of maturation.

Analysis of weight patterns in relation to cardiac disease severity added further nuance to our findings. Across both time periods, children with more severe CHD, including those with Fontan circulation, had markedly lower BMI percentiles and did not demonstrate the same degree of post-lockdown weight gain seen in non-Fontan patients. This observation is expected and aligns with the well-described nutritional, metabolic and physiological challenges associated with complex congenital heart disease. This gradient likely reflects physiological and nutritional challenges characteristic of advanced CHD, including chronic low-output states, increased metabolic demands, impaired gastrointestinal perfusion, feeding difficulties and progressive loss of lean muscle mass, all of which predispose to growth faltering and cachexia. In contrast, children with mild or surgically repaired CHD, whose exercise tolerance and metabolic reserve are relatively preserved, remain exposed to the same obesogenic environments as the general paediatric population and may therefore accumulate excess adiposity. These divergent patterns underscore the need for disease severity specific strategies: supporting growth and preventing undernutrition in those with complex CHD, while emphasising obesity prevention and lifestyle modification in patients with milder lesions.

### Hypertension and its clinical relevance

The prevalence of hypertension in our population was high (34% pre-lockdown and 29% post-lockdown when classified as stage 1 or 2 hypertension). Although blood pressure staging differed significantly when analysed across four categories, this finding was driven by a shift away from stage 2 hypertension rather than a reduction in overall hypertension prevalence. These figures should therefore not be interpreted as true hypertension prevalence. Our definitions were based on single clinic readings obtained using automated devices, which are susceptible to measurement error, anxiety-related “white coat” effects and incorrect cuff sizing. Single readings have been shown to label more children as hypertensive than repeated measurements [19]. In our cohort, the observed reduction in blood pressure staging and blood pressure percentiles post-lockdown is likely to reflect measurement variability, regression to the mean and greater clinical familiarity with paediatric blood pressure assessment over time, rather than a genuine improvement in underlying haemodynamics. This interpretation is further supported by external data; a recent systematic review and meta-analysis reported a global hypertension prevalence of approximately 4% among children and adolescents, far lower than the ~ 30% proportion observed in our sample [20]. This discrepancy strongly suggests that many elevated blood pressure readings in our audit represent measurement artefact or transient elevation rather than sustained hypertension.

Despite this, the consistent positive correlation between BMI and systolic BP in both pre- and post-lockdown periods indicates that excess body weight does contribute meaningfully to higher BP levels, even if the absolute prevalence is overestimated. The stronger correlation post-lockdown suggests that prolonged exposure to obesogenic environments during the pandemic may have intensified this association. The absence of a comparable correlation with diastolic BP is in keeping with prior studies showing that obesity more strongly influences systolic than diastolic BP in children [21, 22].

These findings have important clinical implications. Elevated BP readings, particularly in children who are overweight or obese and those with CHD, should not be dismissed as spurious without further evaluation. Instead, they should trigger repeat manual measurements, ideally in a calm setting with careful attention to cuff size and technique, followed by ABPM where indicated. This stepped approach would help distinguish transient or artefactual elevations from true hypertension and guide appropriate management.

### Clinical implications and service gaps

Children with CHD experience accelerated cardiovascular ageing, characterised by early arterial stiffness, endothelial dysfunction, reduced exercise tolerance and heightened vulnerability to metabolic stressors [9, 15]. Superimposed obesity and sustained hypertension are likely to accelerate this trajectory, leading to earlier onset of heart failure, arrhythmias and ischaemic events. Yet, our audit shows that obesity and hypertension are rarely documented as active problems in paediatric cardiology clinic letters and that referral to weight-management or hypertension services is infrequent.

At present, CHD is not explicitly listed as a comorbidity in referral criteria for the CEW clinic in the UK. Our analysis suggests that adding CHD as an indication would modestly increase the number of eligible patients, but could have a disproportionately beneficial impact by targeting support at those with greatest cardiovascular vulnerability. Extrapolated figures also indicate that a large number of children seen annually in EMCHC clinics meet BP thresholds that should prompt further evaluation, yet very few are referred for specialist hypertension assessment. While this may partly reflect clinician confidence in managing mild obesity or isolated blood pressure elevation, the complexity and vulnerability of this population argue for a lower threshold for specialist input.

Reduced exercise tolerance and fatigue in CHD may make it particularly challenging for affected children to engage in conventional lifestyle interventions. This underscores the importance of early, tailored multidisciplinary support, including dietetic input, physiotherapy or exercise physiology and psychological support. Failure to address these modifiable risk factors risks perpetuating a cycle of deconditioning, weight gain and worsening cardiovascular function. Our findings align with emerging conclusions from the UK COVID-19 Inquiry, which has highlighted widening health inequalities, loss of routine paediatric monitoring and reduced continuity of chronic disease pathways during the pandemic [23]. The significant post-lockdown increase in BMI percentile observed only among South Asian children mirrors national evidence that minority ethnic groups experienced disproportionate health impacts during and after lockdown. Likewise, the under-recognition and undertreatment of obesity and hypertension in our cohort reflect broader system-wide reductions in preventive care capacity and missed opportunities for early intervention. The shift toward non-integrated, private-sector digital weight management services further echoes inquiry concerns regarding inequitable access and fragmentation of care. By quantifying these cardiometabolic effects in a vulnerable paediatric population, our study provides condition-specific insight supporting calls for more resilient, equitable and coordinated long-term paediatric services.

### Impact of CEW decommissioning

The CEW service at our centre has been recently decommissioned, widening the gap in specialist provision for children with severe obesity and cardiometabolic complications. National CEW clinic models remain under evaluation, with economic results pending. In the absence of stable NHS provision, private-sector and digital-health providers, many offering already remote prescription of pharmacological weight loss therapies, are increasingly positioned to fill this gap. These services are not integrated with paediatric cardiology and often emphasise pharmacological interventions over holistic, multidisciplinary care. This risks further fragmentation of services, reduced continuity of care and widening health inequalities for children with complex cardiovascular needs.

### Limitations

This audit has several limitations. Data were collected retrospectively from routine clinical records and may therefore be incomplete or inconsistently recorded. We were unable to verify the accuracy of weight, height or BP measurements nor could we control for factors such as device type, resting time or patient anxiety. BP categorisation was based on a single measurement at each visit, contrary to guideline recommendations for at least three readings, and ABPM was infrequently used. These factors likely contributed to an overestimation of hypertension prevalence. Furthermore, referral data captured only those patients who had attended follow-up appointments; we did not review waiting lists or declined referrals, which may underestimate the true number of children for whom further management was intended.

### Recommendations

Recommendations arising from this audit include integrating BMI and BP percentiles into routine documentation in cardiology outpatient clinics to support more accurate interpretation and timely follow-up. Elevated BP should be confirmed with repeated manual readings and, when indicated, ABPM to avoid misclassification due to measurement variability or white-coat effects. Given their increased cardiovascular vulnerability, children with CHD should be considered explicitly eligible for referral to CEW services. Improving staff training in accurate paediatric BP measurement, including correct cuff selection and repeat measurement protocols, would enhance reliability. Strengthened collaboration between cardiology, general paediatrics and weight-management teams, alongside targeted strategies for high-risk ethnic groups such as South Asian children, may further improve early identification and management of obesity and elevated BP in this population.

## Conclusion

Obesity and hypertension are under-recognised and undertreated in paediatric cardiology patients, despite their substantial contribution to long-term cardiovascular risk. Although many children met hypertension thresholds, this likely reflects measurement limitations and white-coat effects rather than true disease burden. Even so, the strong association between BMI and systolic BP highlights the need for repeated manual BP measurements and ambulatory monitoring to confirm hypertension.

Children with CHD face accelerated cardiovascular ageing, making early identification and management of modifiable risk factors essential. Routine percentile-based assessment, improved BP measurement practices and expanded referral pathways are crucial for improving outcomes. A proactive, multidisciplinary approach has the potential to reduce cardiovascular burden and improve long-term survival in this vulnerable population.

## Data Availability

All data supporting the findings of this study are available within the paper. All data was from a clinical audit (authorisation number 13825) from the University Hospitals of Leicester audit department.

## Appendix 1. Definitions of BMI and blood pressure categories

### BMI categories

BMI-for-age percentiles were used to classify weight status as follows:

- Underweight: < 5th percentile
- Healthy weight: 5th to < 85th percentile
- Overweight: 85th to < 95th percentile
- Obese: 95th to < 99.6th percentile
- Severely obese: ≥ 99.6th percentile

These categories are standardised for age and sex.

### Blood pressure categories (children < 13 years) [11]:

BP percentiles were used to define blood pressure status in accordance with paediatric hypertension guidelines. Classifications were adjusted for age, sex and height:

- Normal: SBP/DBP < 90th percentile
- Elevated BP: SBP/DBP 90th to < 95th percentile *or* 120/80 mmHg to < 95th percentile (whichever is lower)
- Stage 1 hypertension:
  - ⚬ SBP/DBP ≥ 95th percentile to < 95th percentile + 12 mmHg, *or*
  - ⚬ 130/80–139/89 mmHg (whichever is lower)
- Stage 2 hypertension:
  - ⚬ SBP/DBP ≥ 95th percentile + 12 mmHg, *or*
  -  ⚬ ≥ 140/90 mmHg (whichever is lower)

### Blood pressure categories (children ≥ 13 years) [11]:

For patients aged 13 years and older, adult-based thresholds were applied:

- Normal: < 120/80 mmHg
- Elevated BP: systolic 120–129 mmHg and diastolic < 80 mmHg
- Stage 1 hypertension: systolic 130–139 mmHg or diastolic 80–89 mmHg
- Stage 2 hypertension: systolic ≥ 140 mmHg or diastolic ≥ 90 mmHg

## Appendix 2. UHL referral criteria for excess weight and hypertension

### Referral criteria for the complications of excess weight (CEW) clinic

According to University Hospitals of Leicester (UHL) guidelines, the following paediatric patients (aged 1–17 years) should be referred to the CEW clinic:

#### BMI-based criteria

- BMI ≥ 3.33 SDS, irrespective of comorbidities
- BMI ≥ 2.57 SDS (≥ 99.6th centile), or
- BMI ≥ 2.0 SDS (≥ 98th centile) in children of Black, Asian and Minority Ethnic background, plus at least one of the following:
  - ⚬ A comorbidity related to excess weight (e.g. hypertension)
  - ⚬ A confirmed genetic cause of obesity
  - ⚬ A secondary cause of obesity
  - ⚬ Placement on a child protection plan for severe obesity
  - ⚬ Consideration for bariatric surgery

### Hypertension management and referral pathway

UHL guidelines for paediatric hypertension recommend:

#### BP measurement and diagnosis

- Hypertension should not be diagnosed on a single reading.
- BP must be measured on three separate occasions to confirm hypertension.
- All children with elevated BP, pre-hypertension or hypertension should receive lifestyle advice.

#### Referral recommendations

- Children with obesity and confirmed hypertension, who are motivated and have no identifiable secondary cause, should be considered for referral to the CEW clinic.
- All children with suspected secondary hypertension must be referred to a paediatrician experienced in managing childhood hypertension.

#### Severe hypertension

- All patients with stage 2 hypertension should be admitted for monitoring.
- Management must be discussed with a paediatrician specialising in acute and severe hypertension.

## Abbreviations

ABPM: Ambulatory blood pressure monitoring
BMI: Body mass index
BP: Blood pressure
CEW: Complications of excess weight
CHD: Congenital heart disease
DBP: Diastolic blood pressure
EMCHC: East Midlands Congenital Heart Centre
IMD: Index of multiple deprivation
NS: No significant differences
SD: Standard deviation
SBP: Systolic blood pressure
UHL: University Hospitals of Leicester
